# The area-of-interest problem in eyetracking research: A noise-robust solution for face and sparse stimuli

**DOI:** 10.3758/s13428-015-0676-y

**Published:** 2015-11-12

**Authors:** Roy S. Hessels, Chantal Kemner, Carlijn van den Boomen, Ignace T. C. Hooge

**Affiliations:** 1 Department of Experimental Psychology, Helmholtz Institute, Utrecht University, Heidelberglaan 1, 3584CS Utrecht, The Netherlands; 2 Department of Developmental Psychology, Utrecht University, Utrecht, The Netherlands; 3 Brain Center Rudolf Magnus, Department of Child and Adolescent Psychiatry, University Medical Centre Utrecht, Utrecht, The Netherlands

**Keywords:** Eyetracking, Areas of interest, Faces, Sparse stimuli

## Abstract

A problem in eyetracking research is choosing areas of interest (AOIs): Researchers in the same field often use widely varying AOIs for similar stimuli, making cross-study comparisons difficult or even impossible. Subjective choices while choosing AOIs cause differences in AOI shape, size, and location. On the other hand, not many guidelines for constructing AOIs, or comparisons between AOI-production methods, are available. In the present study, we addressed this gap by comparing AOI-production methods in face stimuli, using data collected with infants and adults (with autism spectrum disorder [ASD] and matched controls). Specifically, we report that the attention-attracting and attention-maintaining capacities of AOIs differ between AOI-production methods, and that this matters for statistical comparisons in one of three groups investigated (the ASD group). In addition, we investigated the relation between AOI size and an AOI’s attention-attracting and attention-maintaining capacities, as well as the consequences for statistical analyses, and report that adopting large AOIs solves the problem of statistical differences between the AOI methods. Finally, we tested AOI-production methods for their robustness to noise, and report that large AOIs—using the Voronoi tessellation method or the limited-radius Voronoi tessellation method with large radii—are most robust to noise. We conclude that large AOIs are a noise-robust solution in face stimuli and, when implemented using the Voronoi method, are the most objective of the researcher-defined AOIs. Adopting Voronoi AOIs in face-scanning research should allow better between-group and cross-study comparisons.

Across all fields of research using eyetracking as a research method, areas of interest (AOIs) are used to link eye-movement measures to parts of the stimulus used (e.g., the time spent looking at a particular object in the stimulus). AOI statistics—for example, dwell time (Holmqvist et al., 2011, p. 386)—can make eye-movement data easier to interpret and are used in multiple fields of research, such as user interaction, marketing research, and psychology. Although it is common to provide motivation for which AOIs to construct, it is uncommon to motivate the shape and size of these AOIs. The problem that arises is that AOI statistics from different studies using similar stimuli are difficult to compare. In the present study, we explore the methods and guidelines available for AOI construction, and subsequently evaluate AOI methods for use in face stimuli: a research field in which researchers do not necessarily apply the same AOIs, though the stimuli used are highly similar. This is particularly relevant for researchers investigating, for instance, (the development of) face processing, atypical face processing such as in autism spectrum disorder, and social interaction using eyetracking.

Constructing AOIs, and more specifically choosing the size and shape of these AOIs, can be a difficult choice (see, e.g., Goldberg & Helfman, 2010). Holmqvist et al. (2011, pp. 218–219) describe that when the semantic parts of the stimulus image are clearly discernable—for instance, an airplane’s cockpit with separate panels and dials for separate functions—AOIs are often defined by an expert. An expert may be the researcher involved or an external expert—for instance, a pilot in the previous example of an airplane cockpit. However, even if AOIs are expert-defined and the locations for candidate AOIs are clear, the size and shape of a specific AOI set depend on the specific expert involved in defining the AOIs. One might wonder whether experts in the same field define the same AOIs for an identical or a similar stimulus set. Moreover, constructing a sensible AOI set does not only warrant knowledge of the stimulus semantics (i.e., which parts of a stimulus belong to which AOI), but also of the quality of the data collected. Data quality, and more specifically, the spatial accuracy of an eyetracking study, determines the minimum size of the AOIs needed in order to capture gaze toward that location. Data quality depends on factors such as the eyetracking system and participant group involved in the study, and can affect AOI-based eyetracking measures (Holmqvist, Nyström, & Mulvey, 2012). Finally, AOI production can be a laborious process when it is done by hand. Although there are machine-made alternatives, they are not widely applied (see Table 1). We will explore the methods of AOI construction used in one particular field of study: face-scanning research, in which experts in the same field do not define the same AOIs.

**Table 1:** AOI methods used in face-scanning studies

Study	Method	AOIs of Inner Facial Features	Fixed AOI Shape	White Space Between AOIs
Hunnius & Geuze (2004)	Grid	Eyes and mouth AOIs constructed from multiple grid cells.	No: AOIs are created from several grid cells	No: follows from grid method
Gallay, Baudouin, Durand, Lemoine, & Lécuyer (2006)	Grid & Hand-drawn	**Grid**: no inner feature AOIs constructed from grid cells.	Yes: rectangles	No
		**Hand-drawn**: Eyes and nose/mouth area constructed for hand-drawn analysis.		
Nguyen, Isaacowitz, & Rubin (2009)	Hand-drawn	Eyes, upper lip, lower lip, nose, glabella, forehead, cheek	No	Yes
Senju, Vernetti, Kikuchi, Akechi, & Hasegawa (2013)	Hand-drawn	Left eye, right eye, nose bridge, mouth, nose	Yes: rectangles	No
Oakes & Ellis (2011)	Hand-drawn	Eyes, mouth, nose	Yes: rectangles	No
Tenenbaum, Shah, Sobel, Malle, & Morgan (2013)	Hand-drawn	Eyes, mouth, nose	Yes: rectangles	No
Võ, Smith, Mital, & Henderson (2012)	Hand-drawn	Eyes, mouth, nose	Yes: rectangle (mouth) and ellipses (other AOIs)	Yes
Kano & Tomonaga (2010)	Hand-drawn	Eyes, mouth, nose	No	No
Chawarska & Shic (2009)	Hand-drawn	Eyes, mouth, nose	No	No
Shic, Macari, & Chawarska (2013)	Hand-drawn	Eyes, mouth, nose	No	No
Liu et al. (2011)	Hand-drawn	Eyes, mouth, nose	No	Yes
Wheeler et al. (2011)	Hand-drawn	Eyes, mouth, nose	No	Yes
Falck-Ytter (2008)	Hand-drawn	Eyes, mouth	Yes: rectangles and ellipses	Yes
Wagner, Luyster, Yim, Tager-Flusberg, & Nelson (2013)	Hand-drawn	Eyes, mouth	Yes: rectangles	Yes
Rutherford & Towns (2008)	Hand-drawn	Eyes, mouth	Yes: rectangles	Yes
Wilcox et al. (2013)	Hand-drawn	Eyes, mouth	Yes: rectangles	Yes
Jones, Carr, & Klin (2008)	Hand-drawn	Eyes, mouth	No	Yes, but only at the location of the nose, which is not included as an AOI
Jones & Klin (2013)	Hand-drawn	Eyes, mouth	No	Yes, but only at the location of the nose, which is not included as an AOI

Only studies reporting visual examples of the AOIs used are included. The methods of AOI construction are elaborated in Table 2. Note that both the Voronoi tessellation and the limited-radius Voronoi tessellation AOI methods are absent in the literature. We are not aware of any face-scanning studies applying these methods

In general, AOIs are constructed separately for each study by the researcher determining the most relevant areas of the stimuli. It is, therefore, not surprising that studies using similar stimuli rarely have identical AOIs. One particularly striking example is in face-scanning research: Although all faces have the same main features (i.e., two eyes, a nose, and a mouth), the AOIs in face-scanning studies vary considerably. Table 1 provides an overview of all the studies investigating gaze behavior with face stimuli that have provided visual examples of the AOIs used. Although the eyes, nose, and mouth features are always used as AOIs, rarely are the AOIs identical, except between studies by the same research group. In addition, the AOIs are almost always constructed using the hand-drawn method; only two studies employed (partly) machine-made alternatives. The question that arises is whether the AOI-production method or AOI set used matters for the AOI-based measures used in analyses. If it does not, one might consider applying the method with the least amount of labor involved. If it does, the question arises of which production method or AOI set is preferable in a specific situation or for a specific AOI-based measure.

Several methods of AOI construction are available; we limit the scope to researcher-defined AOIs—that is, the expert-defined AOI category, as described by Holmqvist et al. (2011). Although multiple other methods are available, including data-driven alternatives to AOI analyses (see, e.g., Caldara & Miellet, 2011), we are concerned here with defining AOIs when the features to cover by the AOIs and the hypothesis concerning them are clear. We address additional approaches in more detail in the Discussion section. When considering researcher-defined AOIs, multiple methods for AOI construction exist that differ in a number of ways. AOIs can be implemented fully by the researcher (which we will refer to as *man-made*), or they can be partly implemented by custom software (*machine-made*). Moreover, AOIs can differ in whether they are subjective in location, shape, or size (see Table 2). One approach often used is the manual selection of an area around a part of the stimulus that is of interest to the researcher—for instance, a hand-drawn ellipse around the eye in a static face. Although the specific implementation of creating such AOIs might be limited to certain shapes (e.g., software for constructing AOIs that allows only rectangles and ellipses vs. all forms), this method is most akin to drawing areas on a printout of a stimulus and will therefore be referred to as the *hand-drawn* AOI method. Using this method, the location, size, and shape of an AOI are subjective choices, and the AOIs are man-made. A different method that has previously been used in the face-scanning literature is the grid method. Using this method, a grid is placed over the stimulus and each grid cell is considered an AOI. Subsequently the grid cells can be attributed to features (e.g., column 2, row 1 is part of the “eyes” AOI), but this is not a requirement. Each grid-cell AOI thus has an objective location and shape, yet the AOI size is subjective. The grid method is less laborious to implement than the hand-drawn method; it can be done partly by machine by computing the locations of grid cells, given a cell size. However, *if* one is interested in calculating feature-specific measures (i.e., the total time spent looking at the eyes of a face), the grid method still needs grid cells to be attributed to separate AOIs. If grid cells are attributed to specific AOIs, the location and shape of that specific AOI become subjective choices.

**Table 2:**
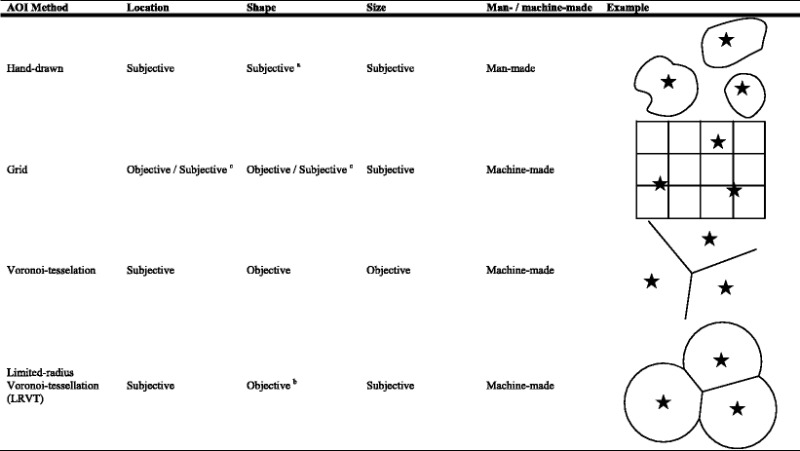
Area-of-interest (AOI) methods with examples in an arbitrary stimulus of three stars

^a^Depending on the implementation of AOI, construction restrictions on shape may occur—for example, custom scripts allowing all shapes versus eyetracker manufacturer software allowing only certain shapes (e.g., rectangles or ellipsoids). These restrictions are at the level of implementation and not at the conceptual level, and therefore will be ignored. ^b^If the grid cells are analyzed as is, and not assigned to specific AOIs, the location and shape are objective. If grid cells are, however, assigned to specific AOIs, a subjective choice has to be made, which makes the location and shape constructed from the grid cells subjective. ^c^Although one might subjectively choose to use the LRVT method and thus the shape of a circle—particularly when there are no Voronoi borders within the chosen radius—the circle shape of the AOI is not based on the stimulus on which the AOI is constructed, and therefore is objective with regard to stimulus content

We present here two related AOI methods based on the tessellation principle introduced by Voronoi (1909), both of which are currently unused in face-scanning research. The first method, the *Voronoi tessellation method* (or the *Voronoi method*, for brevity), can be used to divide an area around a number of points. Each cell defined by the Voronoi method represents the area that is closest to one of the points (i.e., its cell center), and lines indicate locations where any two of the points, or cell centers, are at equal distances. Each fixation that falls within a Voronoi AOI describes that fixation as being closer to its cell center—for example, the center of the nose—than to any of the other cell centers (e.g., the centers of one of the eyes or the mouth). Figure 1 demonstrates an example of Voronoi tessellation with three cell centers. In eyetracking, the Voronoi method has previously been used to quantify distributions of fixations (Over, Hooge, & Erkelens, 2006) and as AOIs for calibration/validation targets (Nyström, Andersson, Holmqvist, & van de Weijer, 2013), and has been applied to many different problems (see Aurenhammer, 1991, for an overview). The Voronoi method includes all of the space of the stimulus, which in turn means that no area of the stimulus is left that does not belong to an AOI. After selection of the cell centers, the construction of AOIs can be done by machine, and both shape and size are objective. A variation of the Voronoi method is what we will call the *limited-radius Voronoi tessellation* method (*LRVT method*, for brevity), the last method we will review. Instead of dividing the stimulus space on the basis of the cell centers, the LRVT method uses cell centers and a given radius to produce AOIs. Each fixation that falls within an LRVT AOI describes that fixation as being both closest to that AOI’s cell center and within a given radius from that cell center. This method could be used if one wanted to use a method similar to the Voronoi method, yet not include all white space (i.e., space that is not part of the stimulus but is included on the screen) around the stimulus. Not including all white space might be useful if much of the gaze path is outside the stimulus of interest—for instance, directed at a cursor that remains on the screen or an object close to the border of the screen. Like the Voronoi method, the LRVT method can be implemented using a machine, yet both the location and radius (and thus the AOI size) are subjective choices. We are not aware of any studies on face-scanning that have used the Voronoi or LRVT method.

**Fig. 1:**
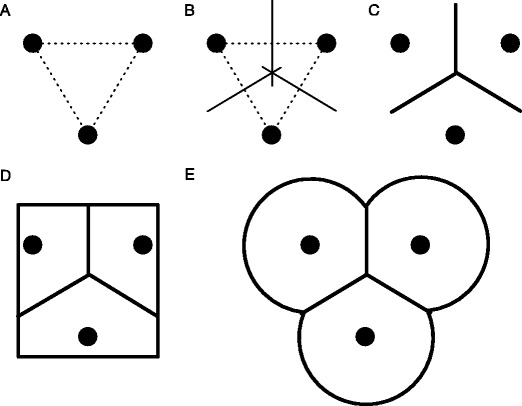
Explanation of the Voronoi tessellation method. **a** Determine the cell centers and draw connecting lines between them. **b** Draw perpendicular bisection lines for each connecting line. **c** Find borders between the cells by connecting the bisection lines. Following Voronoi tessellation, AOIs may be confined—for instance, within the screen or stimulus dimensions **d** or by allowing a maximum radius, as in the limited-radius Voronoi tessellation method **e**. For an elaborate example of Voronoi tessellation using more cells, see Over, Hooge, and Erkelens (2006)

Although no study has directly compared different AOI methods, several researchers have suggested guidelines for AOI construction. For instance, Goldberg and Helfman (2010) suggested that AOIs should only be defined for objects of interest: They need not fill the entire screen. Goldberg and Helfman furthermore suggested that the padding (or margin) around an object should depend on three factors: “(1) the importance of capturing *every* fixation on that object, (2) the amount of white space surrounding the object, and (3) expected variance in fixation positions across participants” (p. 72). Holmqvist et al. (2011) suggested that, when possible, objects of interest should be positioned in the stimulus such that white space can exist between AOIs (i.e., the AOIs should not be contiguous). In addition, they outlined that the minimal AOI size should be determined by the precision and accuracy of the recorded eyetracking data. Finally, Holmqvist et al. suggested that arbitrary AOI positioning should be avoided, but should instead be as precise as possible with regard to the objects of interest in the stimulus. Hooge and Camps (2013) added that for sparse stimuli—relatively empty stimuli in which there is not much crowding (lateral masking), such as faces—the AOIs should be as large as possible. This is because objects of interest are visible from a larger eccentricity in sparse than in dense stimuli. To conclude, AOI construction should depend on both the data quality and the type of stimulus used.

In the present study, we explored how different AOI methods affect eyetracking measures. We evaluated the AOI methods on measures for two AOI characteristics that are commonly used in eyetracking research: attention-attracting and attention-maintaining capacities. These two AOI characteristics were evaluated for the following reasons: We first assume that participants will direct their gaze to AOIs with a high attention-attracting capacity sooner in time than to AOIs with a lower attention-attracting capacity. Attention-attracting capacity might be useful for marketing researchers investigating the time that it takes customers to look at a brand label, or for experimental psychologists investigating reaction times to peripheral targets. The second assumption is that participants will retain their gaze for a longer time in AOIs with high attention-maintaining capacity relative to AOIs with a lower attention-maintaining capacity. Attention-maintaining capacity might, for instance, be useful for developmental psychologists investigating infant preferences for objects or faces.

We focus specifically on three questions regarding the attention-attracting and attention-maintaining capacities of AOIs. (1) How do the attention-attracting and attention-maintaining capacities of AOIs differ between AOI-production methods? (2) What is the relation between AOI size and AOI attention-attracting and attention-maintaining capacity? Are there possible implications thereof for statistical comparisons within and between groups? (3) Which AOI type is most robust to noise? If attention-attracting and attention-maintaining capacities differ between AOI-production methods and are dependent on AOI size, care should be taken when comparing results across studies using different AOIs. In addition, robustness to noise is important to consider when different participant groups are compared in the same study, or when two studies using eyetrackers with different noise levels are compared. In infant eyetracking research, for example, data are typically noisier than in adult eyetracking research. We therefore investigated these three questions in data collected in three different participant groups: typically developing adults, adult with autism spectrum disorder (ASD), and infants. All datasets were obtained using face stimuli, and we will therefore limit ourselves to AOI methods for face-scanning studies. However, because faces are sparse stimuli, our observations might very well generalize to a broader range of studies using sparse stimuli—for example, studies investigating gaze behavior to advertisements or psychological displays, both of which are often sparse. The ASD and typically developing adults, as well as the infant participant group, were chosen because they are of particular relevance in the face-scanning literature. See Guillon, Hadjikhani, Baduel, and Rogé (2014) for a recent review of face scanning in ASD, and, among others, Hunnius, de Wit, Vrins, and von Hofsten (2011) and Wilcox, Stubbs, Wheeler, and Alexander (2013) for face scanning in infancy.

## Method

### Participants

#### Dataset 1—infants

A total of 40 infants (19 male, 21 female) participated in the present study. The mean age of the included group was 311 days (*SD* = 16.7 days). All were born full-term (38–42 weeks) and had normal birth weight, and no delays in development or abnormalities in visual or auditory processing were reported by the health-care system. The medical ethical committee of the University Medical Center Utrecht approved the study. All parents or caretakers gave written informed consent prior to participation and after explanation of the procedure.

#### Dataset 2—ASD and matched controls

A total of 13 young adults with ASD (11 male, two female) and 16 matched control participants (13 male, three female) participated in the experiment. Three of the participants with ASD (two male, one female) were excluded from the analysis either for skipping through more than 20 % of the trials (i.e., making only one fixation, *n* = 2) or because of technical difficulties with the eyetracker (*n* = 1). Five of the control participants (three male, two female) were excluded from the analysis for either skipping through the trials (*n* = 3) or not fixating on the middle of the screen between trials for more than 20 % of the experiment (*n* = 2). Descriptive statistics of the included groups are given in Table 3. For the ASD group, the Wechsler Adult Intelligence Scale III, Dutch edition, was used to determine IQ scores. For the control group, the Wechsler Abbreviated Scale of Intelligence was used to estimate IQ. The diagnostic evaluation for the ASD group included a psychiatric observation and a review of prior records (developmental history, child psychiatric and psychological observations and tests). ASD was diagnosed by a child psychiatrist using the DSM-IV criteria. The medical ethics committee of the University Medical Center Utrecht approved the study.

**Table 3:** Descriptive statistics for the autism spectrum disorder (ASD) and control groups

	ASD Group	Control Group
Sample size	10	11
Age	23.2 (4.25)	23.3 (2.33)
Full-scale IQ	113.3 (9.36)	120.3 (11.67)
Verbal IQ	114.6 (9.96)	121.5 (10.14)

Standard deviations are given in parentheses

### Apparatus and stimuli

An HP EliteBook 8560w was used to present stimuli to a 23-in. external screen (Tobii screen attached to the eyetracker) at a resolution of 1,920 × 1,080 pixels. During the task, eye movements were recorded using the Tobii TX300 at 300 Hz, capable of recording at 0.4° accuracy (binocular) and 0.15° precision (unfiltered) under ideal conditions.^1^

The stimuli consisted of 12 (two expressions and six identities) static pictures of faces taken from the MacBrain Face Stimulus Set.^2^ The pictures were cropped and decolorized (i.e., turned to grayscale pictures). The set contained three female and three male faces, each displaying a neutral and a fearful expression. The stimuli were provided by de Jong, van Engeland, and Kemner (2008). Each face measured 16.7° × 11.5° of visual angle on the screen.

Each face was used in two conditions—high contrast and low contrast—by manipulating the contrast of the eye region. For the infant group, each processed face was presented once, resulting in 24 trials: six neutral low contrast, six neutral high contrast, six fearful low contrast, and six fearful high contrast faces. For the ASD and matched controls group, each processed face was presented eight times, resulting in 192 trials: 48 neutral low contrast, 48 neutral high contrast, 48 fearful low contrast, and 48 fearful high contrast faces. Because the present study was aimed generally at AOIs in face stimuli, the results from the different contrast conditions and emotional expressions are pooled.

### Procedure

#### Dataset 1—infants

The experiment took place either at the infants’ home (*n* = 32) or in the lab (*n* = 8). If the experiment took place at home, a tent was placed over the dining table to approximate equal lighting conditions for each measurement. This tent was specifically designed for conducting research on infants at home. It included fabric in front, to the left and right sides, and above the child to block surrounding visual information. The back of the tent was left open, such that parents or experimenters could be with the child if it felt uncomfortable on its own. For all measurements, the Tobii TX300 was placed on the table, and the infant was placed in a Bumbo seat fitted with a Bumbo Playtray such that the distance between the infant’s eyes and the eyetracker was 65 cm.

The experiment was preceded by a five-point calibration sequence, after which individual points were recalibrated if necessary. The experiment began when the experimenter deemed the calibration sufficient. Each trial began with a colorful movie accompanied by sound, which remained on screen until the infant fixated the screen and the experimenter pressed a button to continue. After this, one of 24 face stimuli was presented for 5 s, after which another movie appeared on screen. The experiment lasted approximately 10 min, including the calibration.

#### Dataset 2—ASD and matched controls

Participants were brought into the lab where they were positioned behind the Tobii TX300. After a built-in nine-point calibration, the experiment began. Each trial began with a black fixation cross, and participants were instructed to press the spacebar to initiate a trial. Next the fixation cross changed color and remained on screen for a variable amount of time (1, 1.25, or 1.5 s). Subsequently, one of the 24 distinct face stimuli was presented, which participants were instructed to look at. A face was presented for a maximum duration of 4 s, or until the participant decided that he or she had seen enough. The experiment for the ASD and matched control groups was self-paced, since we did not want the participants to wonder where to look when they had scanned the stimulus. Participants were not given a task (i.e., free viewing).

For both datasets, the order of pictures was mixed randomly with a set of restrictions using the Mix software (van Casteren & Davis, 2006). Faces of at least two other identities were interleaved between faces of the same identity. In addition, the maximum number of repetitions of the same emotion or the same contrast level was set at three.

### Data reduction

The raw position signals from the left and right eyes were first combined into an average position signal. If gaze position was only available from one eye, that signal was used. Next, a fixation detection algorithm specifically designed for use across varying noise levels—from low noise in the adult data to higher noise in the infant data—was applied. The algorithm operates as an adaptive dispersion algorithm, with which fixation detection can be achieved across larger variations in noise levels, both local and between participants or trials. The algorithm, Identification by 2-Means Clustering (I-2MC), is based on a procedure called *k*-means clustering (where *k* = 2), which is used to determine whether one or two fixation clusters are present in a small moving window. Because the I-2MC algorithm employs a moving window in which clustering is carried out, it is robust to variations in local noise. In the present study, we used a moving window of 200-ms width.

**Fig. 2:**
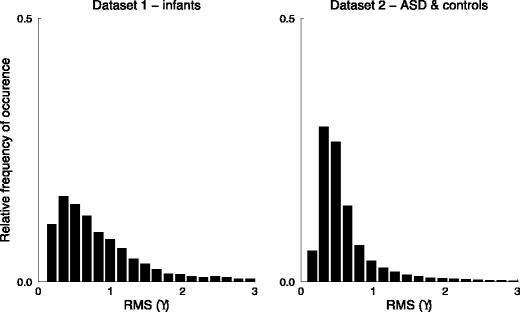
Histograms of the root-mean squared (RMS) noise, in degrees, during fixations for the infant dataset (*left panel*) and for the autism spectrum disorder (ASD) and matched controls dataset (*right panel*)

After event detection, the root-mean squared (RMS) noise for each fixation was calculated as an estimate for the precision of the recording (Holmqvist et al., 2011, p. 35). Although the Tobii TX300 is capable of recording with 0.15° precision, this does not represent the normal value obtained in most eyetracking studies. As can be seen from the histograms in Fig. 2, the distribution of RMS noise in the adult dataset was narrow as compared to the infant dataset. Higher RMS noise was relatively more common in the infant dataset, as is visible from the slightly longer right tail of the distribution.

#### AOI span

In the present study we investigated, amongst other factors, AOI size and AOI robustness to noise. Although these are commonly noted in degrees of visual angle, they are difficult to relate to the stimulus used. Knowing, for example, that the size of an AOI is 2° × 2° is meaningless unless we specify the distance between two AOIs; the information is stimulus- and setup-specific. In order to make interpretation of the results presented here easier—especially when relating the findings to the stimuli used—we present another measure: AOI span. AOI span is the mean distance from each AOI cell center to the cell center of its closest neighbor. For example, if all AOIs in a stimulus are circle-shaped and have a radius of 0.5 AOI, and are positioned at equal distances from each other, the borders of the AOIs connect. If, as another example, the location of a fixation on the mouth were moved upward by 1 AOI span, its new location would now roughly be on the nose. For the present study, AOI span was calculated as follows. The closest AOI for both the left and right eyes was the nose (at a distance of 4.4°). The closest AOI for the nose was the mouth (at a distance of 3.5°), and vice versa. As such, the AOI span was 3.95°. Values of distances relating to the stimulus will henceforth be given in terms of AOI span, with degrees given in parentheses.

#### AOI methods

**Fig. 3:**
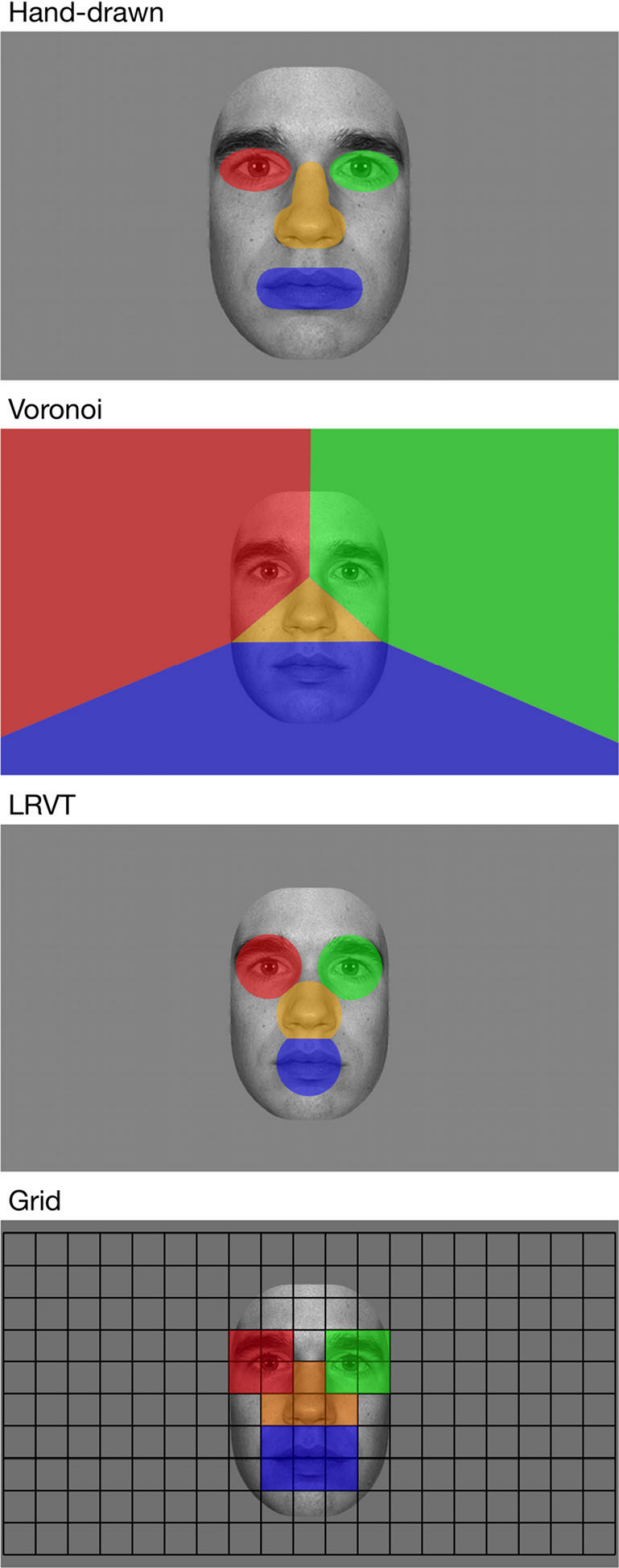
Example of the AOIs used for one stimulus in the present study. Color-coding for the left-eye (*red*), right-eye (*green*), nose (*orange*), and mouth (*blue*) AOIs is maintained throughout the article

Hand-drawn AOIs were manually defined for each individual face using a standard graphics editor (Adobe Photoshop). Areas were defined for the left eye, right eye, nose, and mouth. A non-AOI category was included to capture all gaze data not in the feature AOIs. For the Voronoi AOI method, cell centers were defined in each individual face by determining the center of the pupils, tip of the nose, and center of the mouth. A non-AOI result was included for all gaze data not on the screen (i.e., an infant looking away from the screen). If a fixation was at equal distances from two or more AOIs (i.e., exactly on the border), it was added to the non-AOI results. For the LRVT method, the cell centers from the Voronoi method were used. A non-AOI result was included for all gaze data outside the LRVT radii, as well as for fixations at equal distances from two or more AOIs. We calculated eyetracking measures for the LRVT method with a radius of 0.6 AOI span (corresponding to 2.3°). Although this value is partly arbitrary, the largest differences in eyetracking measures between methods occur for radii around this value. In addition, a 0.6 radius produces AOIs that are just contiguous. We explored the effect of varying LRVT radius in depth in Question 2 of the Results section. The grid method was applied by centering a grid of 19 columns by ten rows (each cell measuring 0.6 × 0.6 AOI span; 2.3° × 2.3°) on the screen (see the bottom of Fig. 3 for an example). Grid cells were subsequently assigned to the left eye (four cells), right eye (four cells), nose (four cells), mouth (six cells), or non-AOI areas. Cells assigned to each AOI were identical across stimuli.

#### Eyetracking measures

As was previously described, we examined the effect of using different AOI methods on two specific characteristics of an AOI: attention-attracting and attention-maintaining capacity. To measure attention-maintaining capacity, dwell time and total dwell time were calculated. *Dwell time* is the time that a gaze remains in a particular AOI, from entry to exit (Holmqvist et al., 2011, p. 386). Dwell time can only be calculated for trials in which time was actually spent in that AOI (i.e., a dwell time of 0 ms cannot occur). *Mean dwell time* reflects the average time that the gaze remained in a particular AOI each single period. AOIs with a higher mean dwell time are assumed to maintain attention for longer individual periods than do AOIs with a lower mean dwell time. *Total dwell time*, on the other hand, is the total time in a whole trial that gaze was in a particular AOI (Holmqvist et al., 2011, p. 389). The total dwell time can be calculated for each trial, regardless of whether an AOI was fixated or not. This means that total dwell times of 0 ms in a trial can also occur. AOIs with a higher mean total dwell time are assumed to maintain attention longer overall than do AOIs with a lower mean total dwell time. To measure attention-attracting capacity, the *time to the first AOI hit* was calculated: that is, the latency from trial onset to the time at which gaze first entered a particular AOI (Holmqvist et al., 2011, p. 437). Note that attention-attracting and attention-maintaining capacities are AOI-specific. Changing the size or location of an AOI results in a different AOI, and the amount of data that it includes changes also. By changing the size or location, a new AOI has been created, with its own attention-attracting and attention-maintaining capacities. Although two AOIs from different AOI methods may share the name (i.e., the “left eye” AOIs for the LRVT and hand-drawn methods), they differ in the amounts of data they include, and thereby in their attention-attracting and attention-maintaining capacities, as estimates for the underlying stimulus feature that they aim to cover.

## Results

### Question 1—how do the attention-attracting and attention-maintaining capacities of AOIs differ between AOI-production methods?

**Fig. 4:**
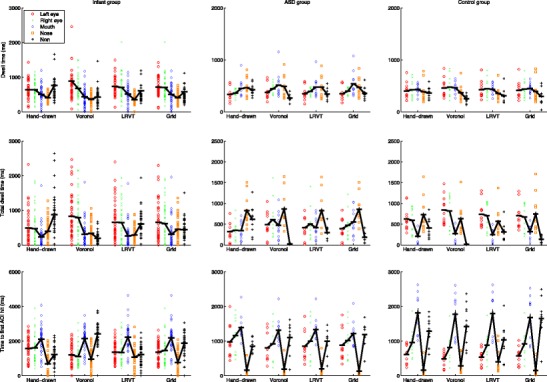
Participant means for dwell time (*top panels*), total dwell time (*middle panels*), and time to the first AOI hit (*bottom panels*), separated by AOI-production methods. Black horizontal bars indicate the group means, and black connecting lines between the AOIs are added to facilitate pattern comparisons across the AOI-production methods. Panels in the left column are for the infant group, panels in the middle column for the ASD group, and panels in the right column for the control group

Raw scores and group means for dwell time, total dwell time, and time to first AOI hit are plotted for each AOI-production method in the left panels of Fig. 4 for Dataset 1 (infants), and in the middle and right panels in Fig. 4 for Dataset 2 (ASD and matched controls). As can be seen from the left panels in Fig. 4, there is some variation in the group means between AOI-production methods for the infant participants. The relative pattern of means to feature AOIs (eyes, nose, and mouth) per AOI-production method is relatively consistent. Mean dwell time and mean total dwell time are consistently longer for the eye AOIs than for the nose and mouth AOIs. Mean time to first AOI hit is consistently shortest for the nose AOI, followed by the eye AOIs and finally the mouth AOI. The (total) dwell times to the non-AOI seem to be inversely related to feature AOI size. This was expected, as larger feature AOIs result in a smaller non-AOI. For instance, they are shortest for the Voronoi method, for which only data outside the screen are labeled as belonging to the non-AOI area. The time to the first AOI hit for the non-AOI region seems related to feature AOI size: the smaller the feature AOIs, the shorter the time to the first hit of the non-AOI.

As can be seen from the middle and right panels in Fig. 4, the patterns observed for the group means between the AOI-production methods for the ASD and matched controls are again consistent. Mean dwell times for the ASD group are consistently slightly shorter for the eye AOIs than for the nose and mouth AOIs for all AOI-production methods. For the control group, the mean dwell times for all feature AOIs are comparable. Mean total dwell times for the ASD group are consistently shorter for the eye AOIs and mouth AOI than for the nose AOI for all AOI-production methods. For the control group, the mean total dwell times are shortest for the mouth AOI, followed by the eye and nose AOIs (although there is some variation in the order of the latter two categories over AOI-production methods). Mean time to the first AOI hit for the feature AOIs is highly consistent over AOI-production methods. For both the ASD and control groups, the order from first to last is nose, left eye, right eye, and finally mouth. There are, however, absolute differences in the means to the first AOI hit between groups. The (total) dwell times to the non-AOI again seem to be inversely related to feature AOI size. They are shortest for the Voronoi method, for which only data outside the screen are labeled as belonging to the non-AOI region. Time to first AOI hit for the non-AOI region seems again related to feature AOI size: the smaller the feature AOIs, the shorter the time to first hit of the non-AOI area. This pattern for times to the first AOI hit as a function of AOI size is, however, less consistent for Dataset 2 than for Dataset 1.

The indication from these results is that although we found some variation between group means across AOI-production methods, the relative patterns remain similar across AOI-production methods, for both the infant and the ASD and matched controls datasets. The absolute differences appear largely due to AOI size, which is further addressed in Question 2. To investigate whether relative differences in total dwell time to AOIs between AOI-production methods affected the statistical outcomes, we carried out paired *t* tests on the mean total dwell times to both eyes (i.e., the sum of the total dwell times to the left and right eyes) and the mouth. This specific comparison was carried out because it relates to a recurring hypothesis with mixed results in the ASD literature (see, e.g., Guillon et al., 2014). Paired *t* tests for both the infant and control participants revealed that for all AOI-production methods, mean total dwell times to the eyes were significantly longer than those to the mouth (*p* < .05). For the ASD group, the mean total dwell time to the eyes was significantly longer than that to the mouth for the hand-drawn, Voronoi, and LRVT AOI-production methods (*p* < .05), but not for the grid method (*p > .*10). This indicates that the differences in eyetracking outcome measures due to the AOI-production method used affected the outcome of a hypothesis-driven experiment. We examined the relationship between AOI size and attention-attracting and attention-maintaining capacities, as well as the effects on our statistical analyses, next.

### Question 2—what is the relation between AOI size and AOI attention-attracting and attention-maintaining capacities?

The relation between AOI size and AOI attention-attracting and attention-maintaining capacities was investigated by applying the LRVT method with a range of radii from 0.05 to 1.52 AOI span (0.2–6.0°) for the feature AOIs. The LRVT method was chosen because it allows for an automated implementation than, for instance, by using increasingly larger hand-drawn AOIs. The maximum of 1.52 (6°) was chosen because this was the mean eye-to-eye difference in the stimulus, and 1.5 times the AOI span. Increasing the LRVT radius results in larger AOIs; hence, LRVT radius is analogous to AOI size. For the non-AOI region, we generally expect the inverse relation of that observed for the feature AOIs: As the size for the feature AOIs increases, the size for the non-AOI area decreases.

**Fig. 5:**
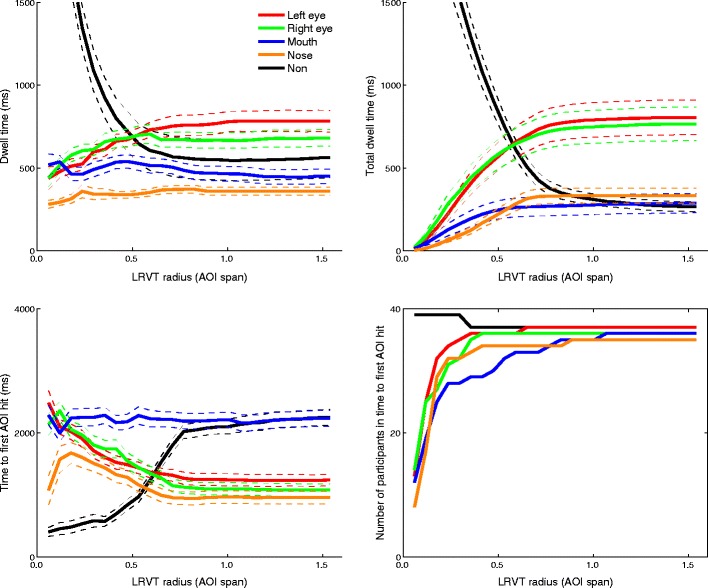
Relation between LRVT radius (given in terms of AOI span) and mean dwell time (*top left*), mean total dwell time (*top right*), and mean time to the first AOI hit (*bottom left*) for the left eye, right eye, nose, mouth, and non-AOI areas for the infant dataset. The numbers of participants for whom a time to first AOI hit could be calculated are depicted in the bottom right panel. Colored dashed lines indicate the standard errors of the means

Figure 5 depicts the relation between mean dwell time, mean total dwell time, and mean time to the first AOI hit and LRVT radius for the infant dataset. As can be seen from the top left panel in Fig. 5, the mean dwell time to the eye AOIs increases with increasing LRVT radius up to around 0.75 AOI span (~3°). For the mouth and nose AOIs, there is no increase in mean dwell time with increasing LRVT radius. The mean dwell time for the non-AOI region decreases up to 0.75 AOI span (3°) LRVT radius. As can be seen from the top right panel in Fig. 5, the mean total dwell time to the feature AOIs increases with LRVT radius, again up to around 0.75 AOI span (3°). Then the mean total dwell times to the feature AOIs do not increase substantially. As expected, the inverse relation was found for the non-AOI region: The mean total dwell time decreases with increasing LRVT radius for the feature AOIs. Note that, unlike dwell times, total dwell times begin at 0. This is expected, because dwell times can only be calculated for trials in which a participant fixated an AOI, whereas total dwell times are 0 when participants do not fixate an AOI.

The mean time to the first AOI hit slightly decreases with LRVT radius for the eye and nose AOIs, whereas it remains relatively stable for the mouth AOI. Mean time to the first hit for the non-AOI increases as the LRVT radius for the feature AOIs increases. The bottom right panel in Fig. 5 depicts the numbers of participants for whom a time to first AOI hit could be calculated. The time to the first AOI hit can only be calculated if an AOI was actually fixated in a trial. As can be seen, the number of participants included in the mean time to the first AOI hit for the feature AOIs increases sharply between 0 and 0.5 AOI span (0°–2°) of LRVT radius.

**Fig. 6:**
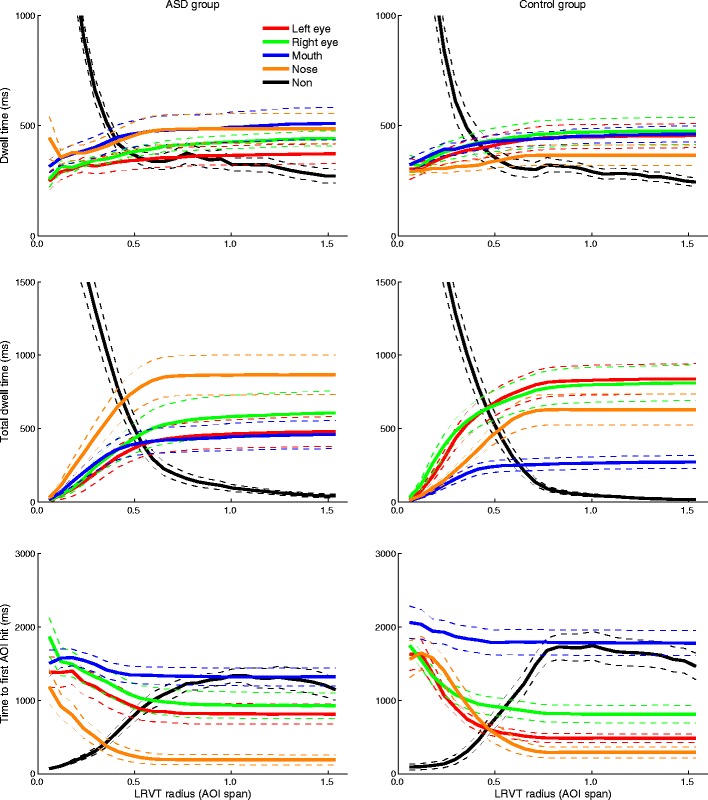
Relation between LRVT radius (given in terms of AOI span) and mean dwell time (*top panels*), mean total dwell time (*middle panels*), and mean time to first AOI hit (*bottom panels*) for the left eye, right eye, nose, mouth, and non-AOI areas. Panels in the left column are for the ASD group, and the panels in the right column for the control group. Colored dashed lines indicate the standard errors of the means. Numbers of participants for whom the time to first AOI hit could be calculated are omitted (as compared to Fig. 5), because almost all participants were included from the smallest radius onward, as a result of more trials being presented

Figure 6 depicts the mean dwell times, mean total dwell times, and mean times to the first AOI hit for all AOIs, separately for the ASD and control groups. The number of participants that were included in the calculation of time to first AOI hit is not depicted, since almost all participants were included from the smallest LRVT radius onward. This was the result of more trials being presented than in Dataset 1.

As can be seen from the top panels in Fig. 6, the mean dwell time to all feature AOIs increases slightly with LRVT radius for both the ASD and control groups, whereas the mean dwell time to the non-AOI area decreases sharply up to 0.75 AOI span (3°) LRVT radius. As is visible from the middle panels in Fig. 6, the mean total dwell time to the feature AOIs increases with LRVT radius but approaches an asymptote after 0.75 AOI span (3°) of LRVT radius. Although the mean total dwell times to the feature AOIs differ between groups, the points at which the increase in total dwell time levels off do not differ between the groups. The mean total dwell time for the non-AOI region decreases sharply up to an LRVT radius of 0.75 AOI span (3°). The mean time to the first AOI hit for all feature AOIs decreases with LRVT radius but approaches an asymptote after an LRVT radius of 0.75 AOI span (3°). The mean time to the first AOI hit for the non-AOI region shows an inverse relation with LRVT radius, and increases up to an LRVT radius of roughly 0.75 AOI span (3°).

**Fig. 7:**
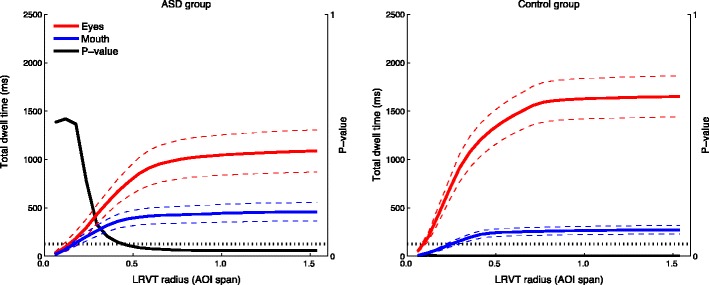
Mean total dwell times to the combined eye AOIs and the mouth AOI for the ASD group (*left*) and the control group (*right*). Colored dashed lines indicate the standard errors of the means. The black lines depict the *p* values of the paired-samples *t* test carried out for each LRVT radius, with *p* = *.*05 being indicated by the dotted horizontal line. Note that the *p* value line for the control group is at 0 for all LRVT radii

**Fig. 8:**
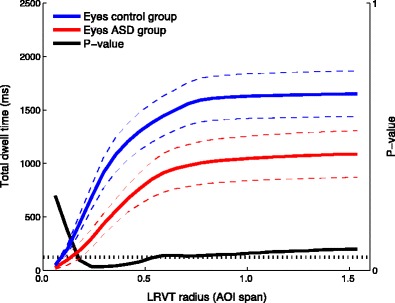
Mean total dwell times to the eye AOIs for the ASD group and the control group. Colored dashed lines indicate the standard errors of the means. The black line depicts the *p* value of the independent-samples *t* test carried out for each LRVT radius, with *p* = *.*05 being indicated by the dotted horizontal line

Following the asymptotic relation between LRVT radius and total dwell time, we examined the effect of LRVT radius on statistical comparisons between AOI attention-maintaining capacities. This was done to examine whether AOI size matters for statistical outcomes. Paired-samples *t* tests were carried out for the comparison of the mean total dwell time to the eye AOIs (sum of the total dwell time to left-eye and right-eye AOIs) and mouth AOI. This was done separately for the ASD and control groups. As is visible from Fig. 7, the difference in total dwell times to the eyes and mouth for the ASD group is significant at *α* = .05 from an LRVT radius of 0.48 AOI span (1.9°) onward. For the control group, however, the difference is significant from the smallest LRVT radius onward. In addition, we examined the between-group comparison of mean total dwell times to the eye AOIs. As is visible from Fig. 8, the independent-samples *t* test on mean total dwell times to the eyes between the ASD and control groups is significant at *α* = .05 between 0.18 and 0.53 AOI span (0.7°–2.1°) of the LRVT radius. Hereafter, the *p* value for the comparison increases slightly, but it remains between .05 and .08 up to 1.5 AOI span of the LRVT radius. We address the implications hereof for research comparing gaze behavior in ASD groups and typically developing controls in the Discussion section.

### Question 3—which AOI type is the most robust to noise?

We investigated the robustness of AOI types to noise by adding Gaussian noise with increasing standard deviations to the fixation locations. Gaussian noise on the fixation location mimics a variable error in the data due to imprecision of the eyetracking measurement. A range of 0.05 to 0.75 AOI span (0.2° to 3.0°) of Gaussian noise standard deviations was added. The reason for the cutoff at 0.75 (3°) is threefold. First, 3° represents three times the size of noise inherent in the worst eyetrackers (see, e.g., Holmqvist et al., 2011). Second, following the LRVT radius analysis (see Question 2 above), the differences in dwell times, total dwell times, and times to first AOI hit were most present between LRVT radii of 0 and 0.75 AOI span. Third, half of the distance between the eyes in the stimulus was 0.75 AOI span. This means that if a fixation were positioned right between the eyes, a horizontal shift between minus and plus one standard deviation would result in a new location anywhere between the left and right eyes.

Robustness to noise was operationalized as the slope of the linear fit between the standard deviations of added Gaussian noise (in AOI span) and the group mean on the eyetracking measure. A slope of 0 would indicate perfect robustness to noise: no increase or decrease in the group means of dwell time, total dwell time, or time to the first AOI hit. The further the slope was away from 0 (whether a positive or negative slope), the less robust to noise the AOI-production method would be.

**Fig. 9:**
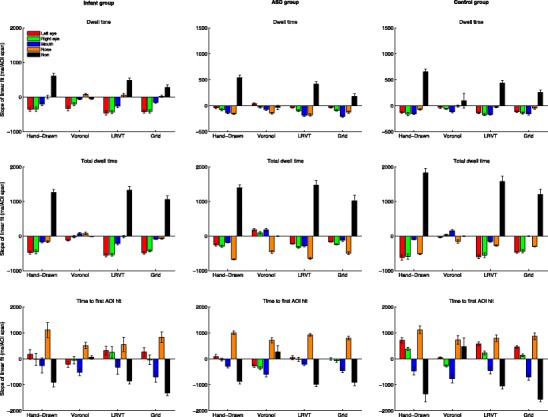
Slopes for the linear fits between the standard deviations of Gaussian noise (°) and the dwell time (*top panels*), total dwell time (*middle panels*), and time to first AOI hit (*bottom panels*). Error bars indicate 95 % confidence intervals of the slopes. Panels in the left column are for the infant group, panels in the middle column for the ASD group, and panels in the right column for the control group

Figure 9 depicts the slopes for mean dwell time, mean total dwell time, and mean time to the first AOI hit versus noise for the infant (left panels), ASD (middle panels), and control (right panels) participants. As is visible from the top left panel in Fig. 9, mean dwell times to the left-eye, right-eye, and mouth AOIs tended to decrease as noise increased for most AOI-production methods. The mean dwell time for the nose AOI remains relatively stable for all production methods, whereas the mean dwell time to the non-AOI area increased for all but the Voronoi AOI-production methods. The fact that dwell time to the non-AOI area does not increase for the Voronoi method is not surprising, because only data outside the screen are labeled as belonging to the non-AOI region. The slopes are closest to 0 for all AOIs for the Voronoi method. As is visible from the middle left panel in Fig. 9, the mean total dwell times decreased for the eye AOIs and the mouth AOI for all but the Voronoi AOI-production method. The mean total dwell time to the non-AOI region again increased for all but the Voronoi AOI-production method as noise increased. Again, the absence of an increase in total dwell time to the non-AOI area for the Voronoi method is not surprising, given that only data outside the screen were labeled as belonging to the non-AOI region. As is visible from the bottom left panel in Fig. 9, the confidence intervals of the slopes for the mean time to the first AOI hit were larger than for the (total) dwell times for all AOI-production methods, indicating that the changes here are less consistent with increasing noise. This might be expected, because the mean time to the first AOI hit is based only on the first fixation in the AOI in each trial. One shifted fixation early in the trial would already result in the measure for that trial being considerably lower. In general, the time to the first AOI hit for the nose increased with increasing noise, and the time to the first AOI hit for the non-AOI region decreased with increasing noise.

As is visible from the middle and right top panels in Fig. 9, the mean dwell times to all feature AOIs were relatively stable across different noise levels for all AOI-production methods. The mean dwell time to the non-AOI region increased as noise increased for the hand-drawn and LRVT methods for both groups. As is visible from the middle panels for the ASD and control groups in Fig. 9, the mean total dwell times to the feature AOIs tended to decrease, whereas the mean total dwell time to the non-AOI region increased with increasing noise. This was, however, much less the case for the Voronoi method in the ASD group, and altogether absent for the Voronoi method in the control group. In the latter case, the confidence intervals of all slopes were closely positioned around 0. As is visible from the bottom panels for the ASD and control groups in Fig. 9, the confidence intervals for the slopes for the mean time to the first AOI hit were relatively large as compared to the confidence intervals for the slopes for mean dwell time and mean total dwell time. The mean time to the first AOI hit tended to increase for the nose AOI, and to decrease for the mouth and non-AOI areas, as noise increased for most AOI-production methods. The slopes for the other AOIs are, however, less consistent over AOI-production methods.

**Fig. 10:**
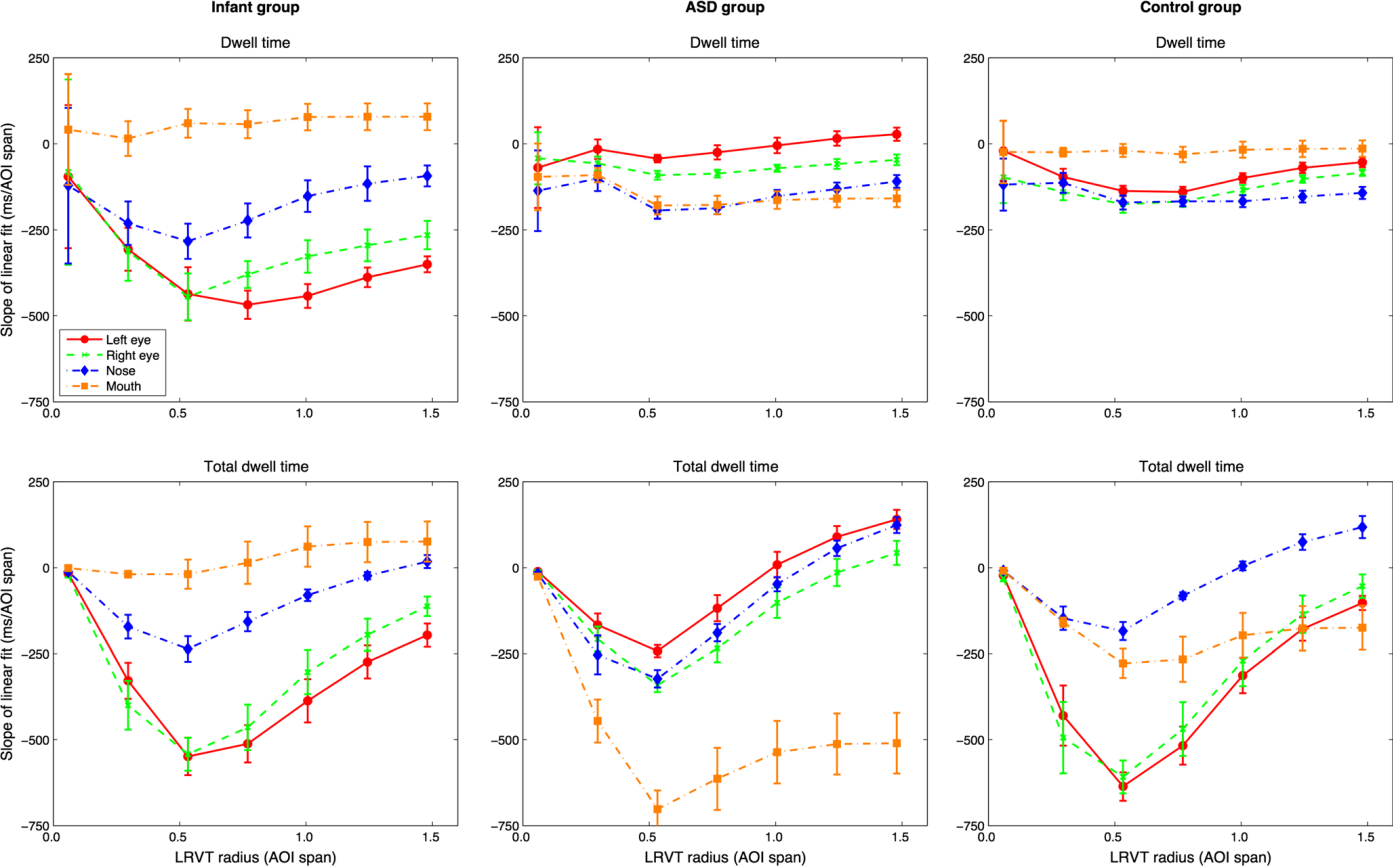
Slopes for the linear fits between the standard deviations of Gaussian noise (°) and the dwell time (*top panels*) and total dwell time (*bottom panels*), as a function of LRVT radius (AOI span). Error bars indicate 95 % confidence intervals of the slopes. Panels in the left column are for the infant group, panels in the middle column for the ASD group, and panels in the right column for the control group

The fact that the slopes are closest to 0 for the Voronoi method, at least for dwell time and total dwell time, led us to further investigate the effects of AOI size on robustness to noise. If the Voronoi method is most robust to noise purely because the AOIs are larger than in the other AOI-production methods, slopes should approach 0 with increasing AOI size. The same approach to AOI size as in Question 2 of the Results section was used to investigate this—that is, by varying the radius of the LRVT AOIs. Figure 10 depicts the slopes for the linear fits between standard deviations of Gaussian noise and dwell times (top panels) and total dwell times (bottom panels) as a function of LRVT radius. As is visible from the top panels in Fig. 10, the slopes for dwell time are close to 0 when the LRVT radius is minimal—very few data are included in the AOIs at this point, see also Question 2 of the Results section—but the absolute slope increases thereafter. The slopes for dwell times approach 0 when the LRVT radius increases beyond 0.5 AOI span (2°), particularly for the infant group, but to a lesser extent for the ASD and control groups. As is visible from the bottom panels in Fig. 10, the slopes for total dwell time are again close to 0 when the LRVT radius is minimal—again, very few data are included in the AOIs at this point—but the absolute slopes increase thereafter. The slopes for total dwell time approach 0 when LRVT radius increases beyond 0.5 AOI span (2°) for all groups. Indeed, larger AOIs are more robust to noise, and especially for total dwell time.

## Discussion

A problem in eyetracking research is choosing AOIs: Researchers in the same field often use widely varying AOIs for similar stimuli, making cross-study comparisons difficult or even impossible. Moreover, subjective choices mean that AOIs differ in shape, size, and location, and whether they are man-made or applied using a computer. However, there are not many guidelines for constructing AOIs or comparisons between AOI-production methods available. In the present study we addressed this by comparing AOI-production methods in face stimuli, using data collected with both infants and adults (ASD and matched controls). Specifically, we report that attention-attracting and attention-maintaining capacities of AOIs differ between AOI-production methods, and that this matters for statistical comparison in one of three groups investigated (the ASD group). In addition, we investigated the relation between AOI size and AOI attention-attracting and attention-maintaining capacities, and the consequences for statistical analyses, and report that adopting large AOIs solves the problem of statistical differences between AOI methods. Finally, we tested AOI-production methods for their robustness to noise, and report that the Voronoi tessellation method is most robust to noise.

We first report that feature AOIs (eyes, nose, and mouth) may differ in size, location, and shape between AOI-production methods. However, the attention-attracting and attention-maintaining capacities of AOIs across AOI-production methods appear to show the same global pattern (e.g., longer total dwell times to the eyes than to the nose for the infant or control group). When tested statistically, the differences between AOI-production methods were large enough, though, to affect the outcome for the ASD group. Using one of four AOI-production methods the difference in mean total dwell to the eyes was not significantly longer than to the mouth, whereas this was the case for the infant and control participants. Because this is a particular relevant analysis in the ASD literature (Guillon et al., 2014), the finding that AOI-production method affects the outcome of statistical tests is not trivial. If the purpose of a study is to compare attention-attracting or attention-maintaining capacity between feature AOIs in ASD, it makes sense to justify the AOIs used in light of the present finding. If, on the other hand, the purpose of the study is to compare attention-attracting and attention-maintaining capacities between feature AOIs for infants or typically developing adults, it should not matter much which AOI-production method is used. It would then make sense to choose the AOI-production method that is most objective and easy to implement. In the present study, the most objective method would be the Voronoi method, of which only the cell centers are subjective. In addition, implementation of the Voronoi method is easy to do by machine once cell centers and fixations have been identified (sample code for the Voronoi and LRVT methods is available from the authors). If, on the other hand, the purpose of a study is to compare the absolute values to feature AOIs to those in other studies, one should take care to construct AOIs using the same AOI-production method for each.

We investigated the relation between AOI size and AOI attention-attracting and attention-maintaining capacities using the LRVT method. LRVT radius was varied between 0.05 and 1.52 AOI span (see the AOI Span section in the Method for details; these measures correspond to 0.2°–6.0°). We report that for both the infant dataset and the ASD and control dataset, the attention-maintaining capacity of feature AOIs increased as a function of LRVT radius, but approached an asymptote around an LRVT radius of 0.75 AOI span (3°). For attention-attracting capacity, the same relation was demonstrated; as LRVT radius increased, so did attention-attracting capacity, up to 0.75 AOI span (3°). Note that an increasing attention-attracting capacity is operationalized as a decreasing time to the first AOI hit. For the infant dataset, the attention-attracting capacities of the feature AOIs were quite inconsistent when the LRVT radius was below 0.5 AOI span (2°). The reason for this was that the number of participants who fixated the feature AOI within the small radius was low for small radii. As the radius increased, so did the number of participants for whom time to the first AOI hit could be calculated. In the adult dataset, in which there were more trials, time to first AOI hit could be calculated for almost all participants from the smallest radius onward. One possible solution for calculating attention-attracting capacity in situations in which only a number of participants fixate a particular AOI is to use the T_50_ (Hooge & Camps, 2013). The T_50_ is the time it takes for 50 % of the participants to hit a particular AOI, which can be calculated instead of a mean over all participants with varying numbers of participants at each data point. The idea behind the T_50_ is that AOIs with a high attention-attracting capacity are assumed to attract gaze more quickly, and for a higher number of participants, than AOIs with a low attention-attracting capacity.

The fact that total dwell times approach an asymptote around 0.75 AOI span (3°) LRVT radius is evidence for fixations being clustered on the stimulus, which is often the case for sparse stimuli. Increasing the size of the AOI no longer includes substantially more data, which means that gaze was directed mostly toward the limited AOI set. If a stimulus were sparse, increasing the size of the AOI would thus mean that more data is included, and differences between or within groups on total dwell time to feature AOIs might be more easily detectable using statistical models. This is also what we report here: If an LRVT radius below 0.48 AOI span were used, one might conclude that the ASD group did not look more toward the eyes than the mouth, whereas for the control group that would be the case. If, on the other hand, an LRVT radius above 0.48 AOI span were used, one would conclude that for both the ASD and control groups, gaze was directed toward the eye region for a longer total period of time than toward the mouth region. Moreover, if no differences between groups were expected within the area not covered by feature AOIs—which in that case would warrant the construction of additional AOIs—it would also make sense to increase the AOI size. If an LRVT radius below 0.18 AOI span were used, one would conclude that the ASD group did not look to the eye region for a shorter total period of time than the control group. If, however, an LRVT radius above 0.18 AOI span were used, one would conclude that the ASD group did, in fact, look to the eye region for a shorter total period of time than the control group. Providing arguments for AOI-production method and AOI size is recommended for future studies. This is particularly relevant for the literature on face-scanning in ASD, where there have been several inconsistent reports as to whether individuals with ASD scan faces differently from typically developing controls (see Guillon et al., 2014, for a review). We suggest using large AOIs when making cross-group comparisons, such that as many data as possible are included.

Finally, the robustness of AOI-production methods to noise was investigated. If the mean values for attention-attracting and attention-maintaining capacities using a particular AOI-production method remain stable across increasing levels of Gaussian noise, this indicates that this method is robust to noise. We report here that the Voronoi method was the most noise-robust method in all three participant groups from two different datasets when attention-maintaining capacity was considered. The reason the Voronoi method is most robust to noise is that its AOIs are largest. Systematically increasing the size of AOIs increases robustness to noise. The attention-maintaining capacity for all AOIs using the Voronoi method remained stable across increasing levels of Gaussian noise with a maximum standard deviation of 0.75 AOI span (3°). This finding is particularly interesting, given recent research. Wass, Forssman, and Leppänen (2014) added Gaussian noise to raw data samples from infant eyetracking data to faces and reported that the distribution of total dwell times across the eyes, nose, and mouth (hand-drawn rectangle shaped AOIs) and the non-AOI region changed. They observed that increased noise was associated with a lower proportion of total dwell time to the eyes, and a higher proportion of total dwell time to the nose, mouth, and non-AOI areas. They concluded that using noncontiguous AOIs might help reduce this error. If we compare their result to the data presented here, we see the same pattern of results for the slopes of mean total dwell time as a function of noise for the hand-drawn AOIs in the infant group. Mean total dwell time to the eyes decreased as a function of noise, decreased much less for the nose and mouth AOIs, and increased for the non-AOI region. If we translate this to a proportion of looking time, we would find a decrease in proportion of the total dwell time to the eyes, and consequently a slightly increased proportion of total dwell time to the nose, mouth, and non-AOI regions. When we consider the Voronoi AOI-production method, we find almost no decrease or increase in mean total dwell times as a function of noise, particularly for the infant and control groups. We should note that the standard deviation of the Gaussian noise (i.e., 3°) added in this analysis was much larger than the error typically reported by eyetracker manufacturers (see, e.g., Holmqvist et al., 2011). Under normal circumstances and when data quality checks are ensured, smaller errors due to noise may be expected than those reported here. Consequently, we argue that adopting large AOIs—most easily implemented using the Voronoi method, optionally extended with a large radius as in the LRVT method—in faces might be a better solution to account for noisy data—for example, in infant eyetracking research—although it should not serve as a replacement for acquiring high-quality data (see, e.g., Hessels, Andersson, Hooge, Nyström, & Kemner, 2015; Nyström et al., 2013).

Adopting large AOIs may seem counterintuitive, given that Holmqvist et al. (2011) suggested that AOIs should be as precise as possible with regard to the objects of interest in the stimulus. However, as Hooge and Camps (2013) already pointed out, for relatively empty stimuli in which there is not much crowding (lateral masking), such as faces, the AOIs should be as large as possible. Here we have shown that large AOIs include all relevant data and are most noise-robust. In addition, large AOIs constructed using the Voronoi method are also easily implemented. Although large AOIs are most suitable for hypothesis-driven research with a clear division of the relevant areas in a stimulus, fine-grained spatial effects cannot be uncovered using large AOIs. As we pointed out above, when such fine-grained spatial effects are hypothesized, they would require additional AOIs to be defined, as opposed to only the main feature AOIs presented here. Moreover, when such fine-grained spatial effects can not be hypothesized in terms of easily distinguishable AOIs, but are of the researcher’s interest, data-driven approaches that statistically compare entire fixation maps may be much better suited (see, e.g., Caldara & Miellet, 2011). Such data-driven approaches have already been adopted for face processing in typical adults (Arizpe, Kravitz, Yovel, & Baker, 2012; Blais, Jack, Scheepers, Fiset, & Caldara, 2008; Caldara, Zhou, & Miellet, 2010), infants (Xiao, Xiao, Quinn, Anzures, & Lee, 2013), and ASD participants (Shi et al., 2015; Yi et al., 2013; Yi et al., 2015). To conclude, the purpose of the research, combined with the stimuli used, should drive the choice of AOI and the analysis type.

### Conclusions and limitations

We report here that the attention-attracting and attention-maintaining capacities of feature AOIs in faces relative to one another do not differ drastically between AOI-production methods. In addition, we conclude that large AOIs using the Voronoi method or LRVT with large radii are the most objective of the researcher-defined AOIs, and that these are the most noise-robust AOI-production methods for use in face stimuli. The Voronoi method is particularly appealing because it can be implemented using a simple computer script, requiring only the coordinates of the AOI cell centers and fixation locations.

We reason here that, because faces are sparse stimuli, adopting larger AOIs using the Voronoi or LRVT method might generally be the preferred method. Other types of sparse stimuli, however, were not investigated in the present study, and we therefore suggest that this advice should be taken with caution. We welcome future research into the effects of AOI-production methods in other sparse as well as dense stimuli, with which we expect other AOI-production methods to thrive.

## Footnotes

1
www.tobii.com/Global/Analysis/Training/Metrics/Tobii_TX300_Eye_Tracker_Accuracy_and_Precision_Test_Report.pdf

2 Development of the MacBrain Face Stimulus Set was overseen by Nim Tottenham and supported by the John D. and Catherine T. MacArthur Foundation Research Network on Early Experience and Brain Development. Please contact Nim Tottenham at tott0006@tc.umn.edu for more information concerning the stimulus set.
